# Identification and Validation of Constructing the Prognostic Model With Four DNA Methylation-Driven Genes in Pancreatic Cancer

**DOI:** 10.3389/fcell.2021.709669

**Published:** 2022-01-11

**Authors:** Guangyu Chen, Junyu Long, Ruizhe Zhu, Gang Yang, Jiangdong Qiu, Fangyu Zhao, Yuezhe Liu, Jinxin Tao, Taiping Zhang, Yupei Zhao

**Affiliations:** ^1^ State Key Laboratory of Complex Severe and Rare Diseases, General Surgery Department, Peking Union Medical College Hospital, Chinese Academy of Medical Sciences and Peking Union Medical College, Beijing, China; ^2^ Department of Liver Surgery, Peking Union Medical College Hospital, Chinese Academy of Medical Sciences and Peking Union Medical College, Beijing, China; ^3^ Clinical Immunology Center, Chinese Academy of Medical Sciences and Peking Union Medical College, Beijing, China

**Keywords:** pancreatic cancer, DNA methylation, nomogram, prognosis, The Cancer Genome Atlas

## Abstract

**Background:** Pancreatic cancer (PC) is a highly aggressive gastrointestinal tumor and has a poor prognosis. Evaluating the prognosis validly is urgent for PC patients. In this study, we utilized the RNA-sequencing (RNA-seq) profiles and DNA methylation expression data comprehensively to develop and validate a prognostic signature in patients with PC.

**Methods:** The integrated analysis of RNA-seq, DNA methylation expression profiles, and relevant clinical information was performed to select four DNA methylation-driven genes. Then, a prognostic signature was established by the univariate, multivariate Cox, and least absolute shrinkage and selection operator (LASSO) regression analyses in The Cancer Genome Atlas (TCGA) dataset. GSE62452 cohort was utilized for external validation. Finally, a nomogram model was set up and evaluated by calibration curves.

**Results:** Nine DNA methylation-driven genes that were related to overall survival (OS) were identified. After multivariate Cox and LASSO regression analyses, four of these genes (RIC3, MBOAT2, SEZ6L, and OAS2) were selected to establish the predictive signature. The PC patients were stratified into two groups according to the median risk score, of which the low-risk group displayed a prominently favorable OS compared with the high-risk group, whether in the training (*p* < 0.001) or validation (*p* < 0.01) cohort. Then, the univariate and multivariate Cox regression analyses showed that age, grade, risk score, and the number of positive lymph nodes were significantly associated with OS in PC patients. Therefore, we used these clinical variables to construct a nomogram; and its performance in predicting the 1-, 2-, and 3-year OS of patients with PC was assessed via calibration curves.

**Conclusion:** A prognostic risk score signature was built with the four alternative DNA methylation-driven genes. Furthermore, in combination with the risk score, age, grade, and the number of positive lymph nodes, a nomogram was established for conveniently predicting the individualized prognosis of PC patients.

## Introduction

Pancreatic cancer (PC) is a lethal solid tumor with a poor prognosis. The amount of estimated new PC cases and deaths both stand in second place in gastrointestinal cancer in the United States (Siegel et al., 2020). Up to now, surgery remains the foundation of curing PC, but the majority (80%–85%) of PC patients who present with unresectable or metastatic tumors lose the chance of surgery (Mizrahi et al., 2020). Whether it is early detection of resectable PC or late diagnosis of unresectable and metastatic PC, a wide range of PC patients require routine chemotherapy including 5-fluorouracil (5-FU)/leucovorin with irinotecan and oxaliplatin (FOLFIRINOX) and gemcitabine with nab-paclitaxel and other multidrug regimens (Conroy et al., 2011; Von Hoff et al., 2013). Besides the progress on surgery techniques and chemotherapy approaches, exploring valid and novel biomarkers is another efficient method to improve the rate of early diagnosis and predict the prognosis of PC patients.

Epigenetic alterations affect gene function via changing organization and dynamics of chromatin, rather than changes in the DNA sequence (Jabłońska and Reszka, 2017). Several epigenetic regulatory mechanisms include DNA methylation, histone modifications, chromatin remodeling, and altered expression levels of noncoding RNAs. DNA methylation is defined as the methyl groups (CH_3_) transfer to the fifth carbon of cytosine in the CpG dinucleotides to form 5-methylcytosine (5-mC). Meanwhile, an increasing number of researches indicated that DNA methylation alterations served a major role in PC. Koutsioumpa et al. found that Lysine (K)-Specific Methyltransferase 2D (KMT2D), which was regulated negatively by double-site CpG methylation-exerted antitumoral function; meanwhile, knockout of KMT2D increased aerobic glycolysis and proliferative rates via regulation of SLC2A3 (Koutsioumpa et al., 2019). Besides, methylation of cell-free DNA (cfDNA) changes from plasma samples can be utilized for early detection in PC. The combination of the methylation of ADAMTS1 and BNC1 was employed to detect the early stages of PC, which were better than CA19-9 (Eissa et al., 2019). However, previous studies have not combined the methylation microarray and RNA-sequencing (RNA-seq) data systematically to detect the specific characteristics in PC. Therefore, screening the DNA methylation-driven genes and identifying the vital biomarkers in PC for prognostic prediction is urgently needed.

In our research, we used the transcriptomic and DNA methylation expression data to filter differentially expressed genes (DEGs) and further detect DNA methylation-driven genes in PC. Then, we conducted the risk model with four screened genes and validated the robustness in the Gene Expression Omnibus (GEO) dataset. Finally, we established a nomogram via the clinicopathologic risk factors and risk score of DNA methylation-driven genes to predict overall survival (OS) in PC. We expect that these candidate genes may help improve the prediction of prognosis for PC.

## Materials and Methods

### Sample Datasets

With the use of RNA-seq data, 430 DNA methylation expression profiles were extracted from The Cancer Genome Atlas (TCGA). The methylation expression levels were calculated by β values (unmethylated to totally methylated). The gene expression profiles of the GSE62452 cohort were acquired from the GEO. A total of 167 RNA-seq data of normal samples were extracted from the Genotype-Tissue Expression (GTEx) Project.

### Screening of Differentially Expressed Genes Between Pancreatic Cancer and Normal Samples

DEGs were screened between 178 PC samples from TCGA and 171 normal samples (4 from TCGA and 167 from GTEx) utilizing the “limma” R package (Ritchie et al., 2015). Threshold criteria the false discovery rate (FDR) < 0.01 and |log_2_ fold change (FC)| > 1 combined.

### Identification of DNA Methylation-Driven Denes

We identified the DNA methylation-driven genes of which mRNA expression levels are a negative relationship with the DNA methylation levels via linear regression analysis. Meanwhile, the DNA methylation status between PC tissues and normal PC tissues was compared utilizing the Wilcoxon rank-sum test (Cedoz et al., 2018).

### Survival Analysis

To assess the relation between DNA methylation-driven genes and OS of PC patients via the Kaplan–Meier (K-M) survival analyses, the survminer package was used to get the optimal cutoff values of each data.

### Construction and Validation of the Predictive Signature

We used the univariate Cox regression analysis, multivariate Cox regression, and the least absolute shrinkage and selection operator (LASSO) binary logistic regression model to filter four DNA methylation-driven genes, and a predictive signature was constructed by the linear combination of the regression coefficients (β). The formula of risk score was below. Risk score = (β1 × expression level of RIC3) + (β2 × expression level of MBOAT2) + (β3 × expression level of SEZ6L) + (β4 × expression level of OAS2). Based on the median cutoff value in TCGA dataset, all PC patients were grouped into two groups of high and low risk. Time-dependent receiver operating characteristic (ROC) curves were displayed for assessing predictive capacity. GSE62452 cohort was utilized to validate the robustness of the prognostic signature.

### Screening the Clinical Factors for Prognosis

To assess the actual clinical significance of predictive signature and other clinical factors that were related to the prognosis of PC patients, we performed a preliminary screening by the univariate Cox regression analysis. The multivariate Cox regression analysis was used to narrow the confounding variables.

### Building the Nomogram

We built a nomogram with every independent prognostic variable. Calibration curves were displayed to assess the predictive power of the nomogram in which the 45° line meant the best prediction.

### Gene Set Enrichment Analysis

Gene Set Enrichment Analysis (GSEA) software was performed to identify the biological pathways between the high- and low-risk groups. *p*-Value < 0.05 was considered to be statistically significant.

### Drug Sensitivity Prediction

We predicted the chemotherapeutic drug sensitivity based on the Genomics of Drug Sensitivity in Cancer (GDSC) database (https://www.cancerrxgene.org/). pRRophetic package was used to estimate the half-maximal inhibitory concentration (IC50).

## Results

### Filtration of Differentially Expressed Genes in Pancreatic Cancer From The Cancer Genome Atlas and Genotype-Tissue Expression Database

**FIGURE 1:**
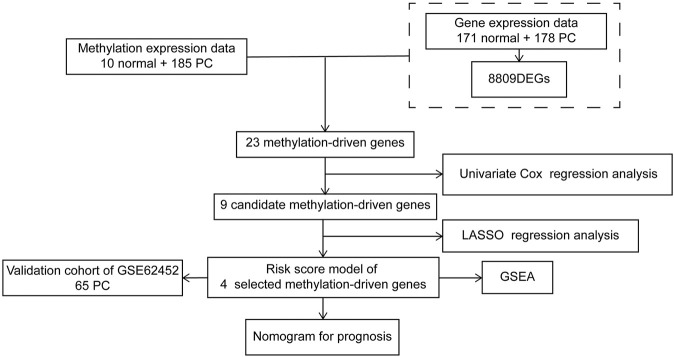
The flow diagram of the research procedure.

**FIGURE 2:**
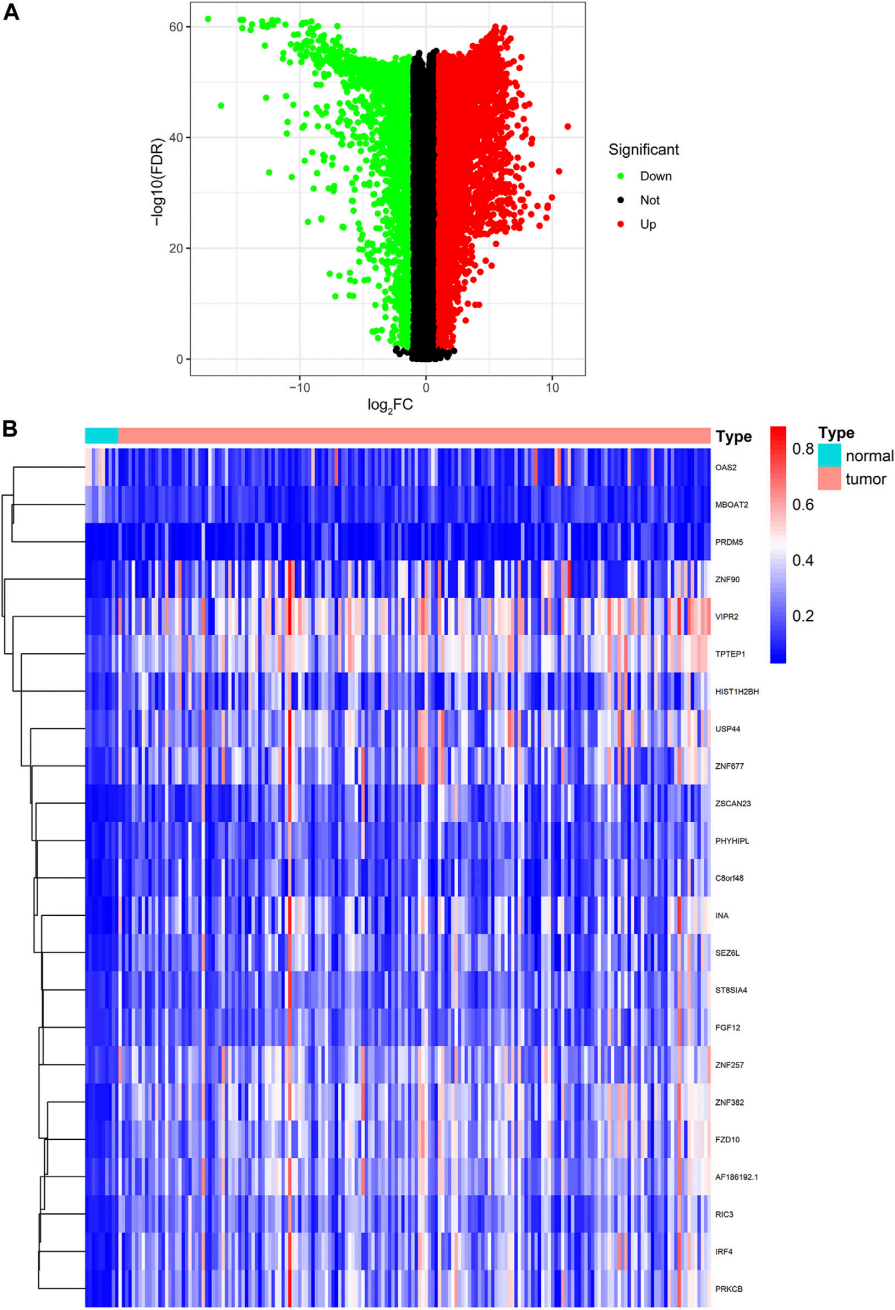
Identification of DEGs and DNA methylation-driven genes. **(A)** Volcano plot of DEGs. **(B)** Heatmap of the methylation levels of 23 candidate DNA methylation-driven genes in PC (*n* = 185) and normal pancreatic tissues (*n* = 10). DEGs, differentially expressed genes; PC, pancreatic cancer.

The flow diagram of this research is presented in Figure 1. The RNA-seq expression data of PC tissues (*n* = 178) and normal pancreatic tissues (*n* = 171, 4 from TCGA and 167 from GTEx) were extracted from TCGA and GTEx database, respectively. After the filtration with cutoff value (|logFC| > 1, FDR < 0.01), 8,809 DEGs were screened for further analysis, including 5,221 upregulated DEGs and 3,588 downregulated DEGs (Supplementary Table S1). The volcano plot is shown in Figure 2A.

### Exploration of DNA Methylation-Driven Genes in Pancreatic Cancer

We utilized the MethylMix analysis to explore DNA methylation-driven genes in PC. Utilizing the screening criteria, we used *p*-value < 0.05 to identify the differentially methylation expressed genes, a correlation <−0.3 was selected between DNA methylation and mRNA expression levels, and a total of 23 DNA methylation-driven genes were screened. The methylation expression level of each DNA methylation-driven gene was visualized via a heatmap (Figure 2B; Supplementary Table S2). Among them, OAS2 and MBOAT2 were hypomethylated; furthermore, the other 21 genes (e.g., PRDM5, ZNF90, VIPR2, TPTEP1, RIC3, and SEZ6L) were hypermethylated in PC.

### Construction of the Prognostic Risk Score Model of DNA Methylation-Driven Genes for Pancreatic Cancer

**FIGURE 3:**
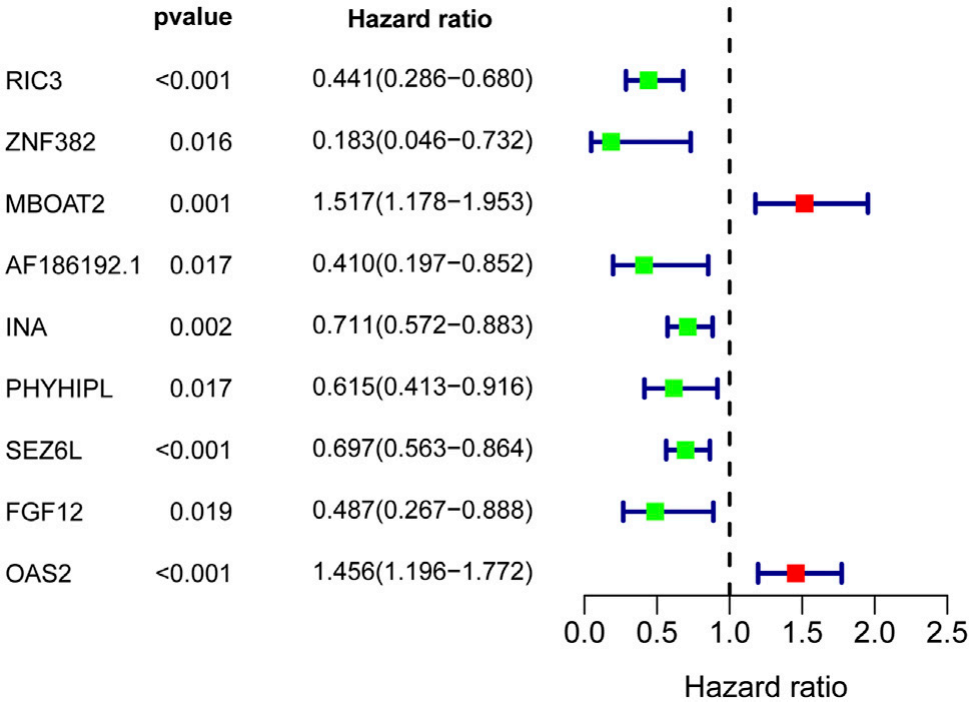
Nine DNA methylation-driven genes were selected by the univariate Cox regression.

**FIGURE 4:**
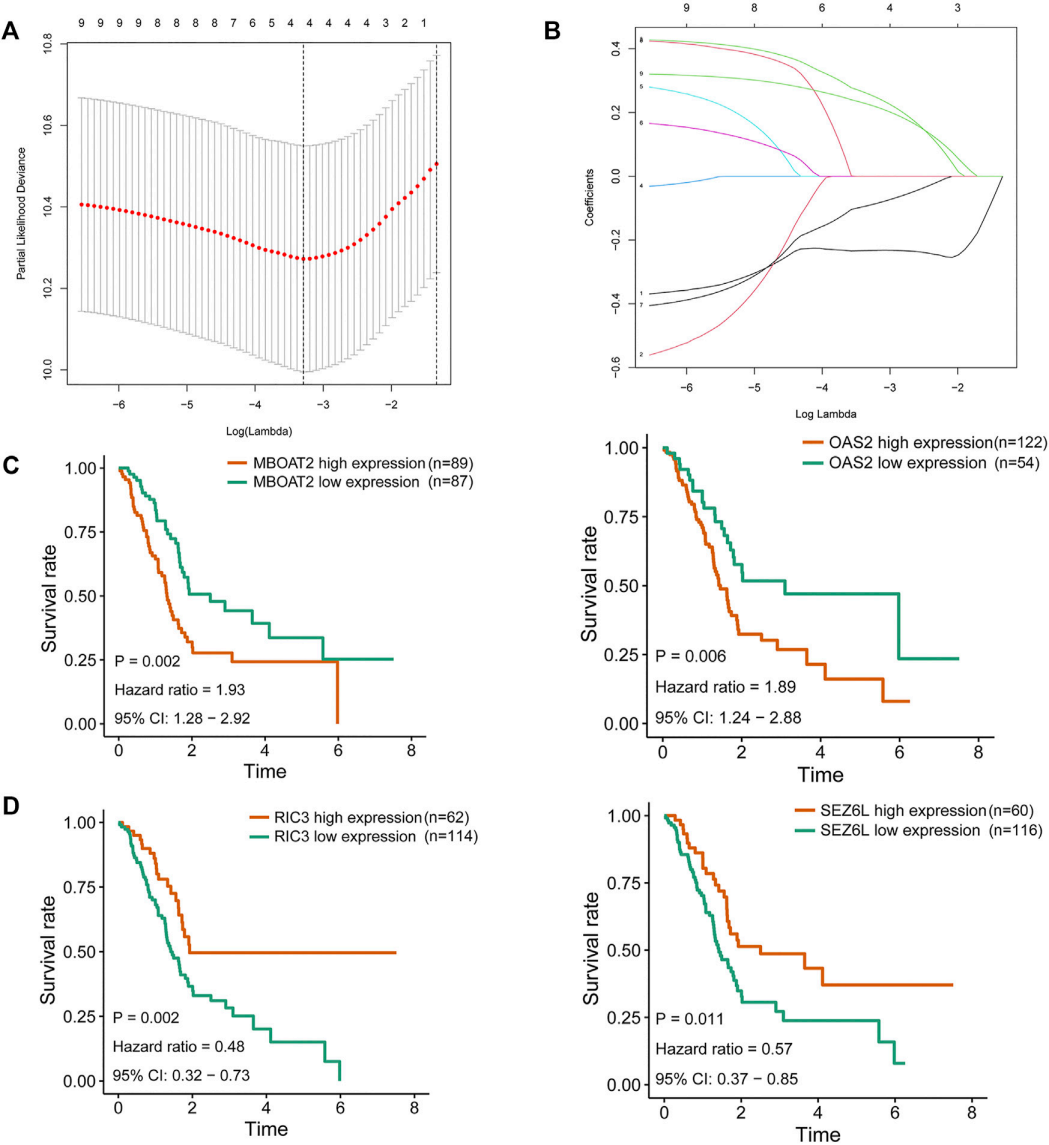
Identification of DNA methylation-driven gene for predictive signature and corresponding survival analysis. **(A)** Filtration of the optimal number of DNA methylation-driven genes by 1,000 iterations of Cox LASSO regression with ten-fold cross-validation. **(B)** Ultimate four genes of nonzero coefficients are selected as candidate DNA methylation-driven genes. **(C)** K-M survival curves for MBOAT2 and OAS2 of which the expression was negatively correlated with OS in PC patients. **(D)** K-M survival curves for RIC3 and SEZ6L of which the expression was positively correlated with OS in PC patients. LASSO, least absolute shrinkage and selection operator; K-M, Kaplan–Meier; OS, overall survival; PC, pancreatic cancer.

**FIGURE 5:**
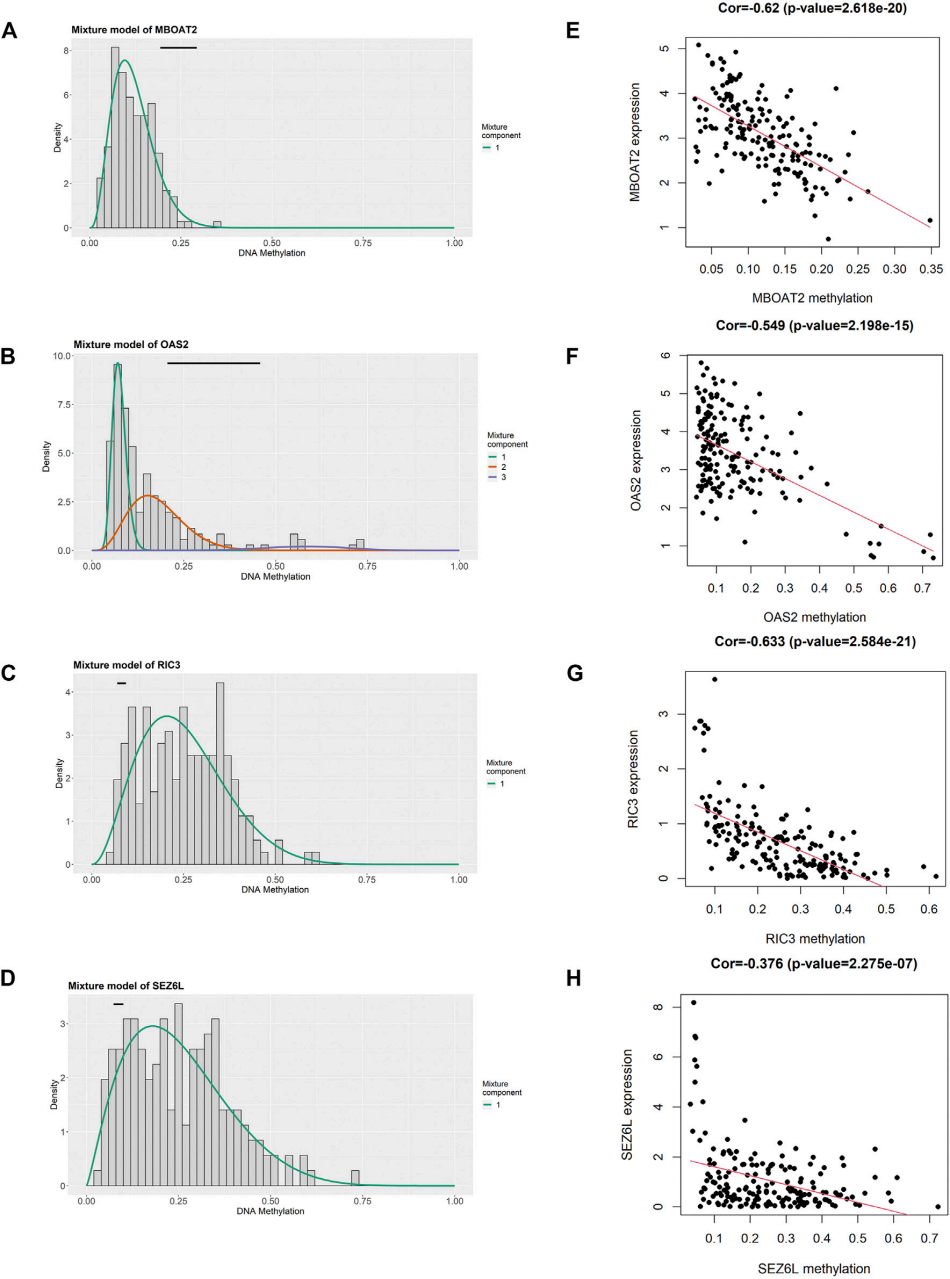
Identification of four DNA methylation-driven genes using MethylMix analysis. **(A–D)** Methylation statuses of four DNA methylation-driven genes. The distribution of MBOAT2, OAS2, RIC3, and SEZ6L methylation in PC samples is displayed by the histogram. The distribution of methylation status in the normal pancreatic samples is displayed by the horizontal black line. **(E–H)** Regression analysis between the DNA methylation level and mRNA expression level of MBOAT2, OAS2, RIC3, and SEZ6L. PC, pancreatic cancer.

**FIGURE 6:**
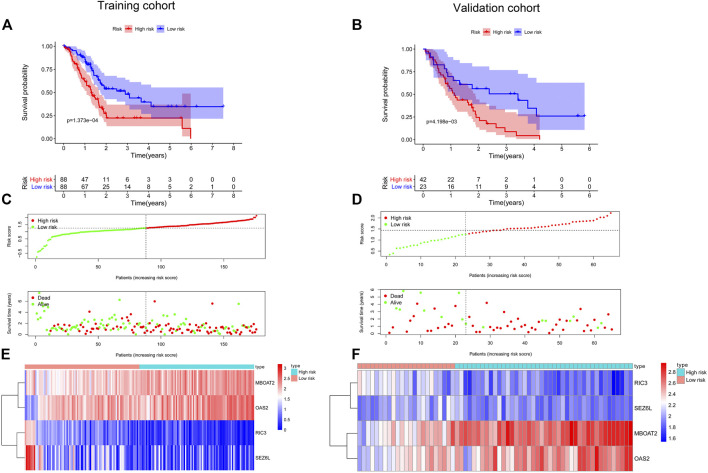
Establishment and validation of a prognostic model for PC. Kaplan–Meier survival curves showed that the high-risk group had shorter OS than the low-risk group in the training cohort **(A)** and the validation cohort **(B)**. The distribution of the survival status and time in different risk groups by the scatter plots in the training cohort **(C)** and the validation cohort **(D)**. Heatmap of the four screened DNA methylation-driven gene expression in the high- and low-risk groups from the training cohort **(E)** and the validation cohort **(F)**. PC, pancreatic cancer; OS, overall survival.

**FIGURE 7:**
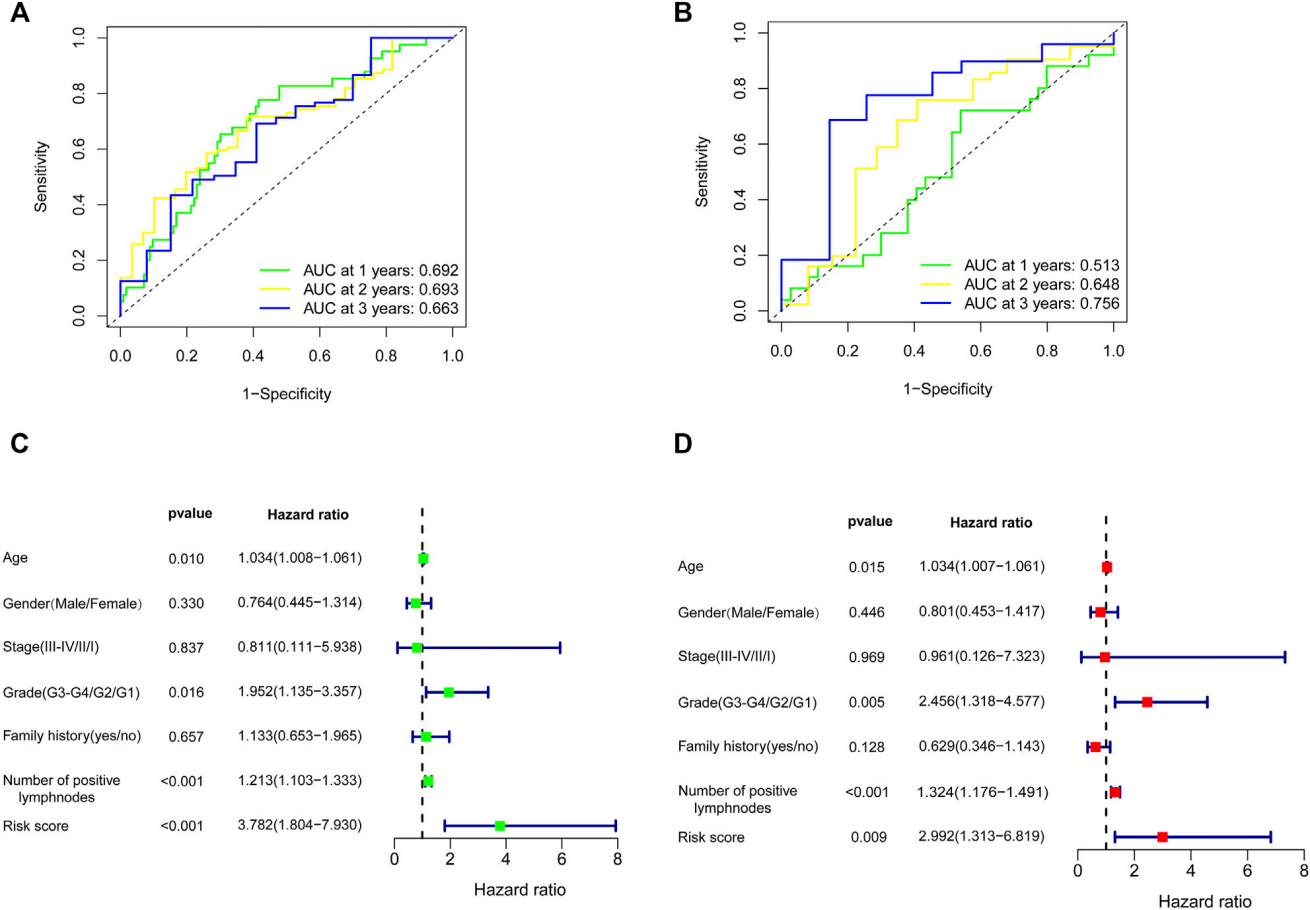
Effects of the risk score model and clinical parameters on the prognosis of PC patients. The time-dependent ROC curve for predicting the 1-, 2-, and 3-year OS rates in TCGA training cohort **(A)** and GEO validation cohort **(B)**. Identification of the parameters related to OS by univariate **(C)** and multivariate Cox analyses **(D)**. PC, pancreatic cancer; ROC, receiver operating characteristic; OS, overall survival; TCGA, The Cancer Genome Atlas; GEO, Gene Expression Omnibus.

To further filter out the candidate genes, we identified the nine candidate DNA methylation-driven genes that were statistically associated with OS (*p* < 0.05) by performing the univariate Cox proportional hazards regression analysis (Figure 3; Supplementary Table S3). LASSO regression analysis is a method that narrows regression coefficients toward zero by an L1 penalty to shrink and select potential variants with nonzero coefficients (Tibshirani, 1997). Moreover, performing 1,000 repetitions of LASSO regression, we found that the variants of nonzero coefficients that occurred more frequently have a stronger capacity to predict prognosis. Finally, the four selected DNA methylation-driven genes (RIC3, MBOAT2, SEZ6L, and OAS2) were selected as prognostic genes by LASSO regression, which were needed to appear 1,000 repetitions and utilized in the risk score model (Figures 4A,B). K-M survival curves of the four selected genes show that the high expression of MBOAT2 and OAS2 had shorter OS than the PC patients of low expression (*p* < 0.01), indicating that MBOAT2 and OAS2 were risk factors in PC (Figure 4C). On the contrary, the high expression of RIC3 and SEZ6L was correlated with a longer survival time (*p* < 0.05), which meant that these genes played a protective role in PC (Figure 4D). Meanwhile, among the four DNA methylation-driven genes, MBOAT2 and OAS2 were hypomethylated, while RIC3 and SEZ6L were hypermethylated (Figures 5A–H). Then, the risk score was calculated with the expression level of each gene multiplied by the relative coefficient in the LASSO regression as follows: risk score = (−0.233 × RIC3 mRNA level) + (−0.079 × SEZ6L mRNA level) + (0.238 × MBOAT2 mRNA level) + (0.211 × OAS mRNA level). Afterward, the risk score of all patients was calculated with the above formula. We selected the median risk score (1.2589) to classify all PC patients into the high-risk group (88 patients) and the low-risk group (88 patients) from TCGA dataset. Meanwhile, the PC patients of the high-risk and low-risk groups were distributed by two distinct patterns by the principal component analysis (PCA) (Figure S1). Meanwhile, the OS of the low-risk group was longer than that of the high-risk group significantly (*p* < 0.001; Figure 6A). The risk scores, survival status, and DNA methylation-driven gene expression profiles of each PC patient were visualized by the heatmap and scatter plot (Figures 6C,E). We also verified the predictive accuracy of the risk model of OS by ROC analysis. The area under the curve (AUC) value of the 1-year OS rate with the prognostic model was 0.692, and the other time-dependent AUC values of 2- and 3-year OS rates were 0.693 and 0.663, respectively (Figure 7A). For further validating the predictive ability of the risk score model, we used the 65 PC samples, which had complete survival information in the validation cohort (GSE62452) from the GEO dataset. Similarly, we utilized the same risk score formula and median cutoff value before and the patients were grouped into two subgroups (the low-risk and high-risk groups). Ultimately, in accordance with the above results, the high-risk group of patients from the validation cohort had a noteworthy worse prognosis than the low-risk group (Figure 6B). The scatter plot of the distribution of risk scores and survival status and the heatmap plot of each gene expression are shown in Figures 6D,F. The AUCs of 1-, 2-, and 3-year OS rates with the prognostic model for PC patients were 0.513, 0.648, and 0.756, respectively (Figure 7B).

### Establishment and Evaluation of a Predictive Prognostic Nomogram for Pancreatic Cancer

We used the univariate and multivariate Cox regression analyses to assess the independent predictive capacity of the four-gene prognostic risk score model in 103 PC patients who possess complete clinical information including age, gender, stage, grade, family history, and the number of positive lymph nodes from TCGA cohort. The results showed that the risk score and clinical factors of age, grade, and the number of positive lymph nodes were correlated with OS by the univariate Cox regression analysis; meanwhile, these factors were also independent prognostic factors associated with OS after the multivariate Cox regression analysis (*p* < 0.05). However, gender, stage, and family history were irrelevant with OS with univariate and multivariate Cox regression analyses (Figures 7C,D).

**FIGURE 8:**
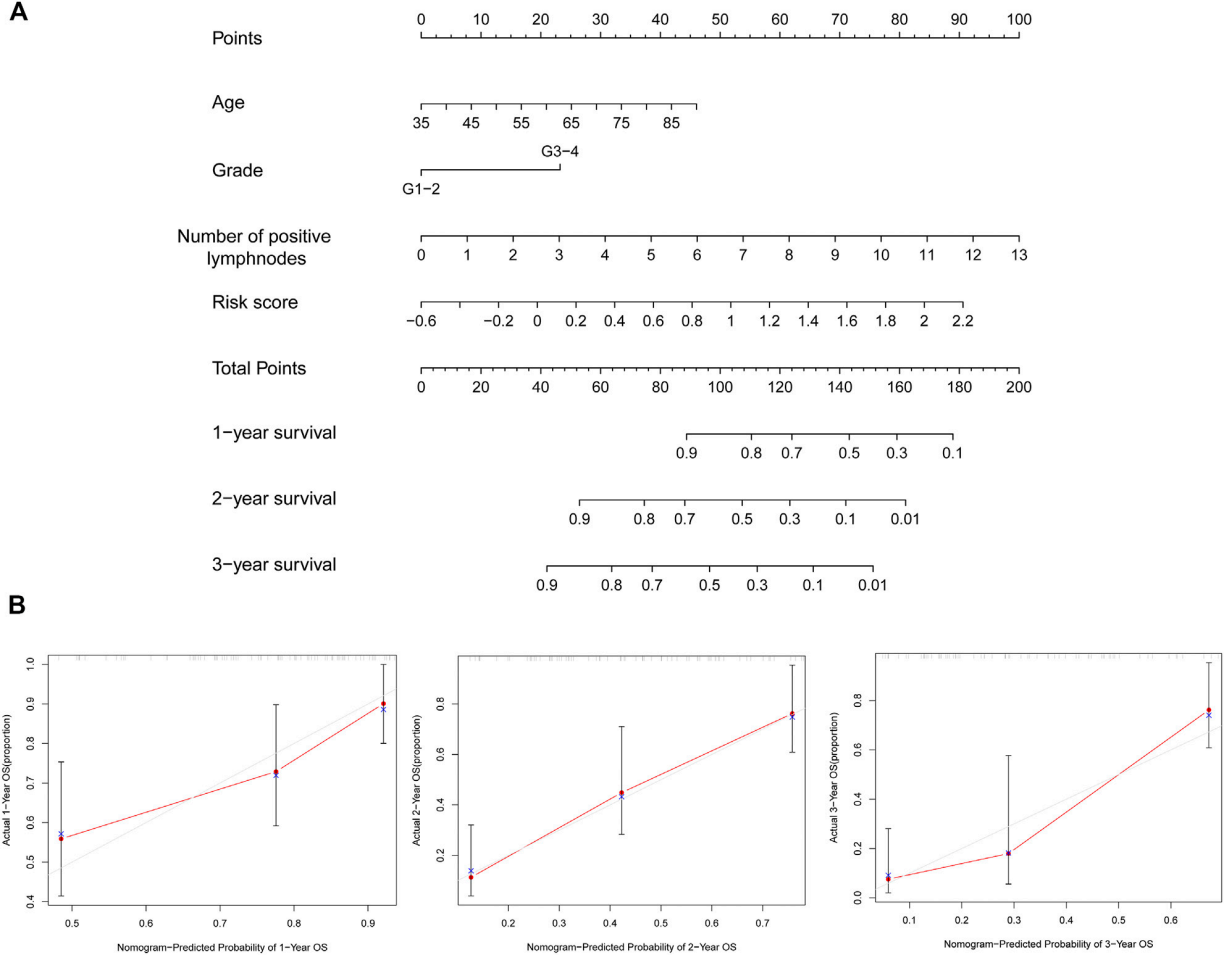
Generation of the nomogram together with prognostic risk signature and clinical parameters. **(A)** Nomogram that combined the risk score and screened clinical parameters to predict the probability of 1-, 2-, and 3-year OS of patients with PC. **(B)** Calibration curves of 1-, two- and 3-year OS were used to evaluate the predictive performance of the nomogram. The 45° line represents the ideal best predictive model. The red line represents the actual model we constructed. OS, overall survival; PC, pancreatic cancer.

Because age, grade, the number of positive lymph nodes, and risk score were considered as significant and independent prognostic factors according to the abovementioned results, we generated a predictive nomogram with these factors (Figure 8A). Besides, the calibration curves of the model were used to evaluate the accuracy of the nomogram in which a 45° line represented the best prediction. As shown in Figure 8B, the predictive OS rates of 1, 2, and 3 years with nomogram demonstrated accurate predictive capacity.

### Gene Set Enrichment Analysis and Drug Sensitivity Prediction Between High- and Low-Risk Groups

**FIGURE 9:**
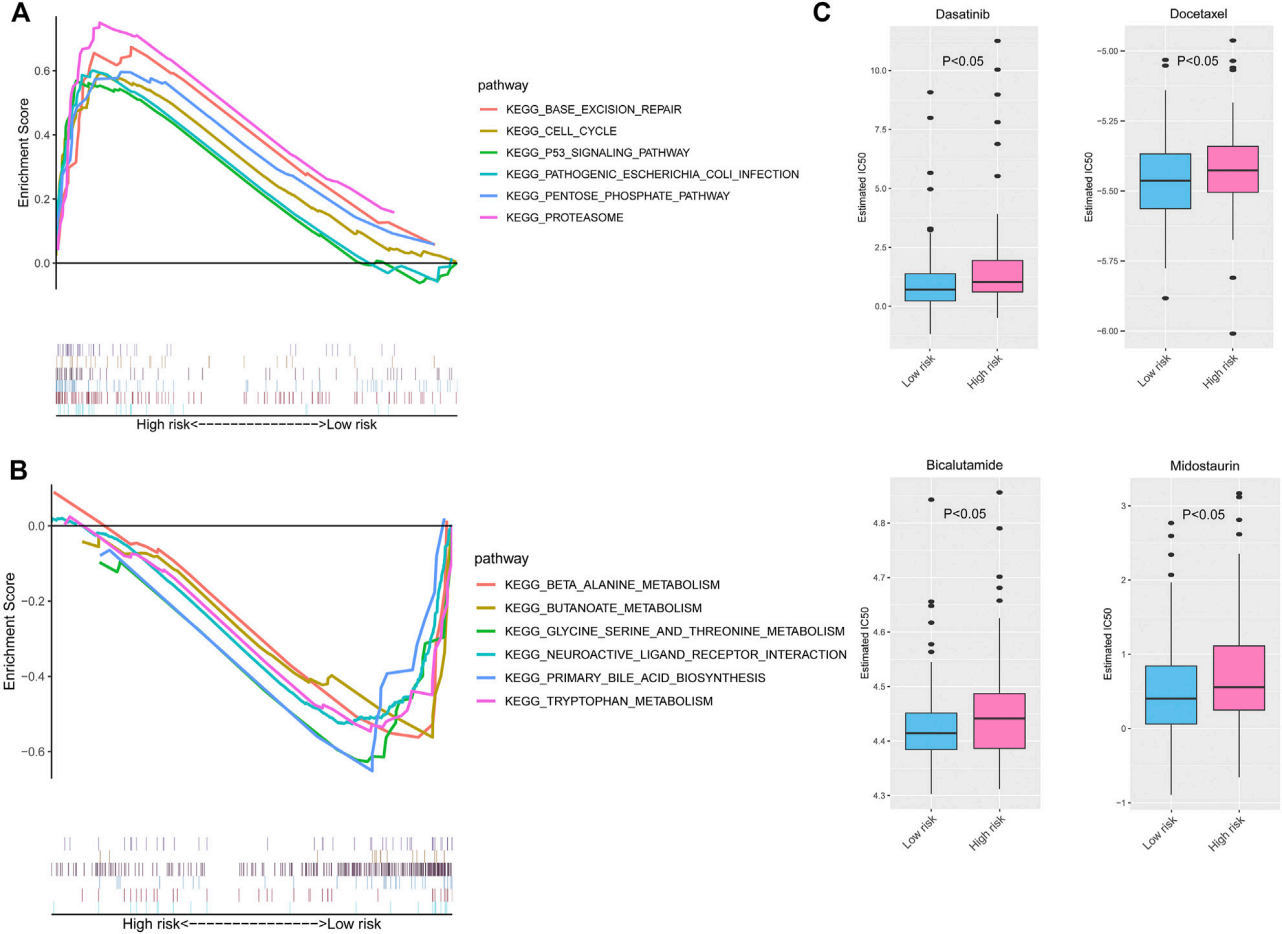
GSEA and drug sensitivity prediction of the risk signature in PC. The enriched KEGG pathways in the high- **(A)** and low-risk groups **(B)** by GSEAs. **(C)** The estimated IC50 of chemotherapy response between the high- and low-risk groups. GSEA, Gene Set Enrichment Analysis; PC, pancreatic cancer; KEGG, Kyoto Encyclopedia of Genes and Genomes.

GSEA was carried out to further explore the possible biological signaling pathways involved with the molecular mechanisms in the risk score model between the high-risk group and low-risk group. As shown in Figure 9A, the top six signaling pathways enriched in the high-risk score group were “BASE EXCISION REPAIR,” “CELL CYCLE,” “P53 SIGNALING PATHWAY,” “PATHOGENIC ESCHERICHIA COLI INFECTION,” “PENTOSE PHOSPHATE PATHWAY,” and “PROTEASOME”. Furthermore, the top six signaling pathways of the low-risk group were significantly enriched for “BETA ALANINE METABOLISM,” “BUTANOATE METABOLISM,” “GLYCINE SERINE AND THREONINE METABOLISM,” “NEUROACTIVE LIGAND RECEPTOR INTERACTION,” “PRIMARY BILE ACID BIOSYNTHESIS,” and “TRYPTOPHAN METABOLISM” (Figure 9B). In general, the enriched signaling pathways in the results of GSEA may indicate significant molecular targets and mechanisms of PC.

Then, we used the GDSC database to predict the valid chemotherapy drugs of high- and low-risk groups. Figure 9C shows that the PC patients in the low-risk group sensitively responded to four drugs (dasatinib, docetaxel, bicalutamide, and midostaurin), whose IC50 was higher than that of the high-risk group (all *p* < 0.05).

## Discussion

PC is one of the most lethal malignancies and has a poor prognosis (Mizrahi et al., 2020). Notwithstanding the benefits from the present progress on the diagnosis and treatment, the 5-year survival rate of PC only rose from 5% to 9% in the past decade (Jemal et al., 2010; Siegel et al., 2020). Up to now, there is a lack of valid and specific approaches to predict prognosis focused on PC patients. As shown in the previous studies, PC is characterized by multiple alterations in the genetic and epigenetic levels (Cancer Genome Atlas Research Network, 2017; Shen et al., 2018). Several studies identified that the innovative DNA methylation markers CD1D, BNC1, and ADAMTS1 have potential for detecting PC (Yi et al., 2013; Kisiel et al., 2015). Meanwhile, Henriksen et al. demonstrated that they established a diagnostic prediction model with eight methylated genes of which AUC is 0.86 for the diagnosis of PC (Henriksen et al., 2016). Besides, the methylation status of three mucin genes (MUC1, MUC2, and MUC4) was used to construct the predicted models for outcome after surgery (Yokoyama et al., 2020). Therefore, identifying specific DNA methylation-driven genes is much vital for PC.

For identifying the global DNA methylation patterns in PC, we combined RNA-seq with DNA methylation profiles to perform a comprehensive analysis. First of all, we screened 8,809 DEGs between PC and normal pancreatic samples from TCGA and GTEx dataset and identified 23 DNA methylation-driven genes by the MethylMix algorithm. Then the univariate Cox and LASSO regression analyses were performed to get four DNA methylation-driven genes (RIC3, MBOAT2, SEZ6L, and OAS2), which were strongly associated with OS. Moreover, the above four genes had been reported in some researches to be closely related to the different types of tumors. Resistance to inhibitors of cholinesterase 3 (RIC3) was identified to fuse with T-cell receptor beta constant 2 (TCRBC2) as fusion transcript and considered carcinogenic in T-cell lymphoblastic lymphoma (Ló pez-Nieva et al., 2019). Loss of the normal function of seizure-related 6 homolog like (SEZ6L) could accelerate the progression of lung cancer (Gorlov et al., 2007). Suzuki et al. elaborated that SEZ6L was hypermethylated and might be involved in the development of colorectal cancer (Suzuki et al., 2002). Besides, CpG methylation of SEZ6L was increased in gastric cancer compared with non-neoplastic mucosa and played a carcinogenic role (Kang et al., 2008; Sepulveda et al., 2016). 2′-5′-Oligoadenylate synthetase 2 (OAS2) had increased expression and was screened to construct prognostic signature in oral squamous cell carcinoma (OSCC); meanwhile, the high expression of OAS2 was associated with poor OS (Wang et al., 2020).

Next, based on these four DNA methylation-driven genes, we constructed the risk score model to evaluate the prognosis of PC, and the patients were distinguished into high- and low-risk groups, which demonstrated a distinct distribution by PCA. Of note, high-risk patients had a shorter OS than low-risk patients, whether in the training cohort from TCGA dataset or the validation cohort (GSE62452) from the GEO dataset. Then the time-dependent AUCs of 1-, 2-, and 3-year OS rates were used to assess the accuracy of the risk score model, and the results displayed good predictive ability. Furthermore, we filtered several clinical information (age, grade, and the number of positive lymph nodes) as independent prognostic factors associated with OS and built a nomogram with the risk score and these clinical factors to predict the individual possible survival times in clinical practice. The calibration plots indicated that the nomogram had an excellent and credible predictive property. To further study which biological mechanisms play vital roles in different risk groups, we conducted GSEA, and the results show that the pathways of p53 signaling, cell cycle, base excision repair, *Escherichia coli* infection, and proteasome were significantly enriched in the high-risk group. However, the pathways related to amino acid metabolism and neuroactive ligand–receptor interaction were significantly enriched in the low-risk group. Furthermore, PC is frequently resistant to chemotherapy, which leads to a poor prognosis. Our results showed that the patients in the low-risk group might benefit from multiple drugs including dasatinib, docetaxel, bicalutamide, and midostaurin. It means that the risk model based on the DNA methylation-driven genes might be a guide for chemotherapy regimens for PC patients.

As far as we know, the predictive model with four DNA methylation-driven genes has not been previously reported in PC, and it will be useful for evaluating the prognosis of patients with PC from a clinical perspective. Furthermore, there are also some limitations in our study. Though some research had proved that the expression or DNA methylation levels of several genes in our prognostic signature were correlated with prognosis in many cancers, the related functions of four genes were not demonstrated in PC. Besides, the risk score model in our present study displayed favorable performance in TCGA dataset and external validation, but there is a lack of evidence to confirm that our predictive signature is preferable to traditional testing methods, such as imaging evaluation or CA19-9. Thus, we are required for further biological experiments to verify the specific roles of four genes in PC. In addition, the nomogram incorporates age, grade, the number of positive lymph nodes, and risk score to predict the OS of PC patients successfully, but the number of PC patients and other clinical characteristics was insufficient. Afterward, much more sequencing data and clinical information from the multi-centric study are indispensable, and a better prognostic nomogram will be constructed in the future.

In conclusion, a risk score model of four DNA methylation-driven genes and the nomogram were built and have reliable predictive capacity for PC. In clinical practice, measuring the expression levels of four genes to calculate the risk score and integrating it with age, grade, and the number of positive lymph nodes of PC patients can prompt individualized prediction of OS in PC patients.

## Data Availability

The datasets presented in this study can be found in online repositories. The names of the repository/repositories and accession number(s) can be found below: https://www.ncbi.nlm.nih.gov/geo/, GSE62452.
