# Supplementary figures and images for: Identification and Validation of Constructing the Prognostic Model With Four DNA Methylation-Driven Genes in Pancreatic Cancer
Source: Front Cell Dev Biol. 2022 Jan 11;9:709669. doi: 10.3389/fcell.2021.709669 (PMC8786741; doi:10.3389/fcell.2021.709669)

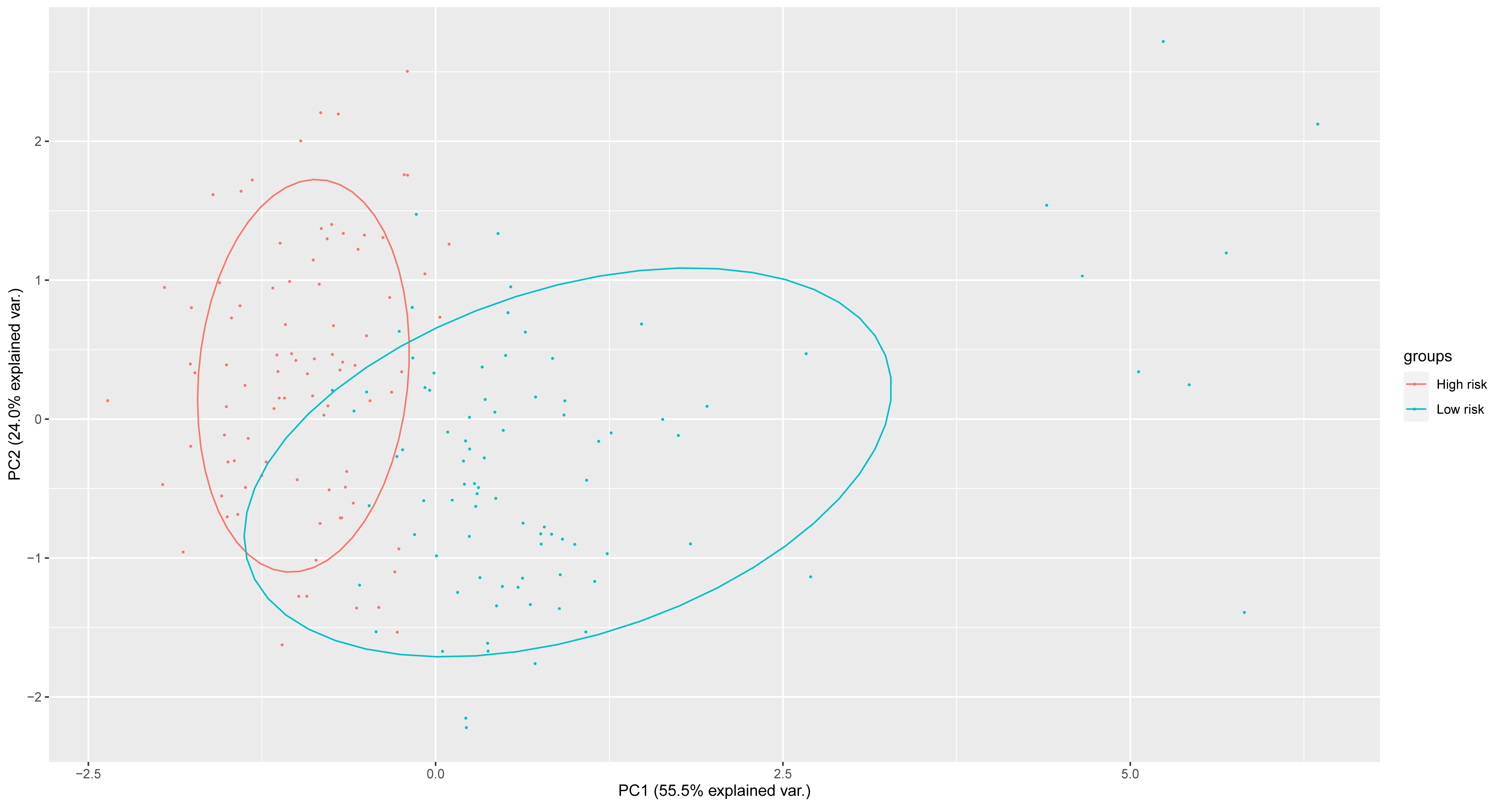

Supplement: Supplementary file 3 [file Image1.JPEG]
