# Supplementary material for: Identification and Validation of Constructing the Prognostic Model With Four DNA Methylation-Driven Genes in Pancreatic Cancer
Source: Front Cell Dev Biol. 2022 Jan 11;9:709669. doi: 10.3389/fcell.2021.709669 (PMC8786741; doi:10.3389/fcell.2021.709669)
Supplement: Supplementary file 4 [file Table3.DOCX]

Table S3 Univariate cox regression analysis of prognostic DNA methylation-driven genes

| Gene | P value | HR | Lower 95% CI | Upper 95% CI |
| --- | --- | --- | --- | --- |
| RIC3 | 0.000206 | 0.441061 | 0.286282 | 0.679522 |
| ZNF382 | 0.016349 | 0.182933 | 0.045720 | 0.731953 |
| MBOAT2 | 0.001249 | 1.516676 | 1.177725 | 1.953178 |
| AF186192.1 | 0.016831 | 0.409915 | 0.197300 | 0.851648 |
| INA | 0.002036 | 0.710858 | 0.572291 | 0.882974 |
| PHYHIPL | 0.016804 | 0.615294 | 0.413225 | 0.916175 |
| SEZ6L | 0.000976 | 0.697279 | 0.562765 | 0.863945 |
| FGF12 | 0.018981 | 0.487393 | 0.267366 | 0.888488 |
| OAS2 | 0.000179 | 1.456009 | 1.196237 | 1.772191 |

HR, hazard ratio; CI, confidence interval.
