# Supplementary material for: Identification and Validation of Constructing the Prognostic Model With Four DNA Methylation-Driven Genes in Pancreatic Cancer
Source: Front Cell Dev Biol. 2022 Jan 11;9:709669. doi: 10.3389/fcell.2021.709669 (PMC8786741; doi:10.3389/fcell.2021.709669)
Supplement: Supplementary file 5 [file DataSheet1.docx]

**Supplementary Figure Legends**

**Supplementary Figure 1.** Principal component analysis (PCA) based on the high- and low-risk group indicated that two obvious distributions.

**Supplementary Table 1.** A list of 8809 DEGs between the PC and the normal pancreatic samples.

**Supplementary Table 2.** A list of the methylation expression level of each DNA methylation-driven gene in the PC and the normal pancreatic samples.

**Supplementary Table 3.** Univariate cox regression analysis of prognostic DNA methylation-driven genes.
